# Melamine–isatin tris Schiff base as an efficient corrosion inhibitor for mild steel in 0.5 molar hydrochloric acid solution: weight loss, electrochemical and surface studies†

**DOI:** 10.1039/d3ra00357d

**Published:** 2023-06-26

**Authors:** Ifzan Arshad, Khizar Qureshi, Awais Siddique Saleemi, Ali Abdullah, Aboud Ahmed Awadh Bahajjaj, Shafaqat Ali, Awais Bokhari

**Affiliations:** a Department of Chemistry, University of Management and Technology Sialkot Pakistan mifzan@gmail.com arshadmi@mail.ustc.edu.cn; b Nano & Micro FT Laboratory, Program of Physics, University of Management and Technology Sialkot Campus Sialkot 51310 Pakistan; c Department of Chemistry, College of Science, King Saud University Riyadh 11451 Saudi Arabia; d Department of Environmental Science, Government College University Faisalabad Faisalabad 38000 Punjab Pakistan; e Department of Biological Science and Technology, China Medical University Taichung 40402 Taiwan; f Sustainable Process Integration Laboratory, Faculty of Mechanical Engineering, Brno University of Technology 2896/2 61600 Brno Czech Republic

## Abstract

In the current study, 3,3′,3′′-((1,3,5-triazine-2,4,6-triyl)tris(azaneylylidene))tris(indolin-2-one) (MISB), which is the condensation product of melamine (triazine) and isatin, was investigated as a mild steel corrosion inhibitor in 0.5 M HCl. The ability of the synthesized tris-Schiff base to suppress corrosion was evaluated utilizing weight loss measurements, electrochemical techniques and theoretical computation. The maximum inhibition efficiency of 92.07%, 91.51% and 91.60% was achieved using 34.20 × 10^−3^ mM of MISB in weight loss measurements, polarization, and EIS tests, respectively. It was revealed that an increase in temperature decreased the inhibition performance of MISB, whereas an increase in the concentration of MISB increased it. The analysis demonstrated that the synthesized tris-Schiff base inhibitor followed the Langmuir adsorption isotherm and was an effective mixed-type inhibitor, but it exhibited dominant cathodic behavior. According to the electrochemical impedance measurements, the *R*_ct_ values increased with an increase in the inhibitor concentration. The weight loss and electrochemical assessments were also supported by quantum calculations and surface characterization analysis, and the SEM images showed a smooth surface morphology.

## Abstract 2

In the current study, 3,3′,3′′-((1,3,5-triazine-2,4,6-triyl)tris(azaneylylidene))tris(indolin-2-one) (MISB), which is the condensation product of melamine (triazine) and isatin, was investigated as a mild steel corrosion inhibitor in 0.5 M HCl.

## Graphical abstract

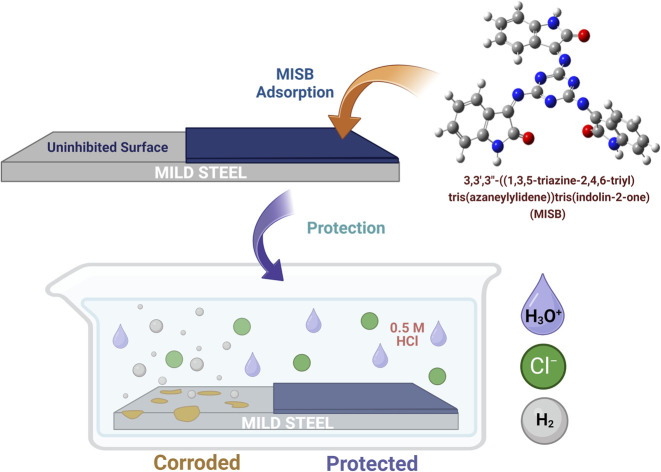

## Introduction

Mild steel is very cheap and it possesses excellent mechanical strength, making it a popular choice in the petroleum and natural gas industries. In this case, strong acids, such as hydrochloric acid, are utilized for acid descaling, acid pickling, and oil well acidification to eliminate the undesirable salt deposits and scales to improve oil recovery.^1^ However, strong acids may corrode the surface of mild steel, ultimately resulting in expensive repairs and system maintenance and financial, physical, and environmental losses.^1,2^

Organic corrosion inhibitors are the most common type of corrosion inhibitors,^3^ which are categorized based on their chemical structure, mechanism of action, and other properties.^4,5^ Their affordability, ease of application, and high degree of protection all contribute to their increasing popularity. They adsorb on the surface of metals and protect them from corrosion,^6,7^ and their effectiveness in a range of acidic solutions is also due to the heteroatoms they contain, such as nitrogen, oxygen, phosphorus, sulfur and halogens.^8–10^ The adsorption of organic molecules on the surface of metal prevents direct contact between the metal and corrosive environment.^11,12^ However, many organic corrosion inhibitors are costly and harmful to human health and the environment. In an acidic environment, organic molecules containing heterocyclic and aromatic heterocyclic rings exhibit greater corrosion inhibition.^5,13–18^ The adsorption of organic molecules is influenced by both chemical and physical bonding. The effectiveness of organic inhibitors can be attributed to their low electronegativity and great polarizability, which allow them to cover huge metal surfaces and quickly transfer electrons to vacant atomic orbitals.^19^ The triazine ring-containing compound known as melamine has three nitrogen atoms, and therefore it is a nitrogen-rich molecule.^20,21^ These nitrogen atoms are readily protonated, thus increasing the solubility of melamine in polar solvents. Recently, substantial progress has been made in the application of melamine derivatives for a range of objectives, including the prevention of corrosion. The remarkable inhibition efficiency of melamine derivatives is attributed to the adsorption of their protonated sites and sharing of their electrons and lone pair electrons with the iron atom.^20,21^ Also, presence of nitrogen and oxygen atoms makes isatin derivatives very efficient corrosion inhibitors among the hetero-atom-containing compounds, and thus several isatin derivatives have been discussed in depth as promising corrosion inhibitors for metals.^22–24^ Recent research showed a tremendous increase in the use of Schiff base derivatives as corrosion inhibitors for metals including steel, aluminum, and copper in very acidic environments.^25–29^ Schiff bases can be easily prepared using extremely cheap starting materials and have little toxicity; therefore, they are becoming increasingly popular as corrosion inhibitors.^30^

The aforementioned factors encouraged us to synthesize a melamine–isatin Schiff base and to evaluate the thermodynamic factors affecting its adsorption, namely, 3,3′,3′′-((1,3,5-triazine-2,4,6-triyl)tris(azaneylylidene))tris(indolin-2-one) (MISB), on the surface of mild steel in an acidic environment using weight loss measurements, electrochemical techniques, surface morphology and computational studies.

## Experimental

### Materials and sample preparation

Chemicals and solvents of analytical grade were used to synthesize the tris-Schiff base of melamine with isatin. All the solvents, including melamine and isatin, were purchased from Sigma-Aldrich and used without further purification. 0.5 M HCl solution was prepared in deionized water using analytical-grade hydrochloric acid (37%). The mild steel specimens used in this work mainly contained the elemental composition of C (0.17%), Mn (1.6%), P (0.040%), Si (0.59%), and Fe (remaining portion). For the weight loss measurements, a specimen with the dimensions of 2 cm × 2 cm × 0.3 cm was used. Prior to the studies, the steel specimen was polished with 600–1200 grade emery paper under a running tap. Finally, it was cleaned with deionized water, and then degassed with acetone and alcohol, followed by drying with cold air. Before the tests, the polished mild steel specimens were kept in a vacuum desiccator.

### Synthesis and characterization data of MISB

**Fig. 1:**
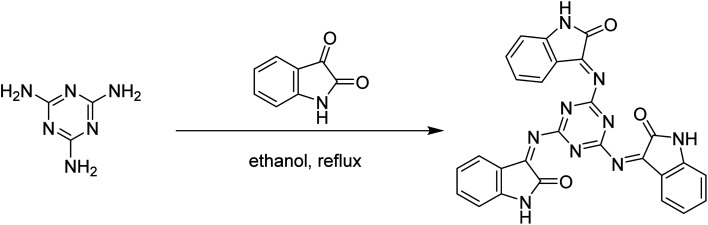
Synthetic scheme for MISB inhibitor molecule.

The Schiff base 3,3′,3′′-((1,3,5-triazine-2,4,6-triyl)tris(azaneylylidene))tris(indolin-2-one) (MISB), as shown in Fig. 1, was synthesized *via* the reaction between melamine with 3 mol of isatin in ethanol as the solvent under reflux, yielding 87% MISB, and its purity was determined by TLC.

#### 3,3′,3′′-((1,3,5-Triazine-2,4,6-triyl)tris(azaneylylidene))tris(indolin-2-one) (MISB)

Yield: 87%, elemental analysis: calc.: C, 63.16; H, 2.94; N, 24.55; found: C, 63.10; H, 2.89; N, 24.41%; characteristics IR peaks (ATR): *ν* N–H = 3502, *ν* C–H (aromatic) = 3053, *ν* C=O = 1631, *ν* C=N = 1592 cm^−1^; ^1^H NMR (400 MHz, DMSO, *δ*, ppm) *δ* = 10.02 (s, 3H, NH), 9.21 (ddd, 3H, Ar–H), 7.69 (ddd, 3H, Ar–H), 7.45 (ddd, 3H, Ar–H), 7.29 (ddd, 3H, Ar–H); ^13^C NMR (100 MHz, DMSO, *δ*, ppm) *δ* = 168.7 (C=O), 162.5 (C=N, triazine), 151.1 (C=N, imine), 148.4 (C–NH), 122.5, 131.9, 128.4, 128.2, 110.0.

### Weight loss measurements

Because of its outstanding accuracy, simplicity, and excellent repeatability, the weight loss method was used to examine the corrosion protection behavior initially. The weighed metallic specimens with dimensions of 2.0 × 2.0 × 0.3 cm were allowed to corrode in 50 mL of 0.5 M HCl without and with varying concentrations of MISB for an immersion time of 4 h in a thermostatically controlled water bath. These specimens were taken out after a particular duration, and the corrosion products on them were gently wiped off with water and acetone. To ensure the reproducibility of the measurement, the weight loss experiment was carried out in triplicate for each investigated concentration of MISB. The following equations were used to determine the rate of corrosion and inhibition efficiency (IE%).^1,31^1
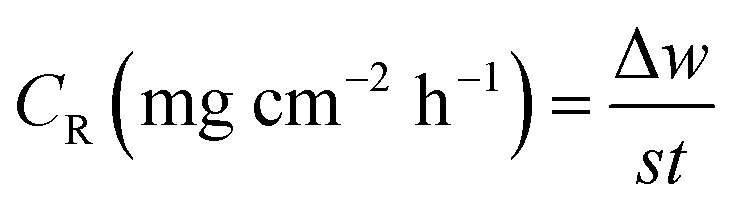
2
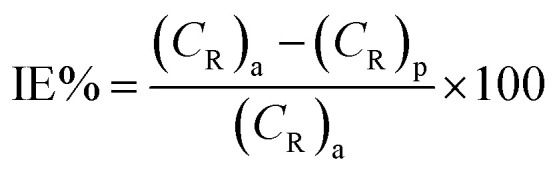
3
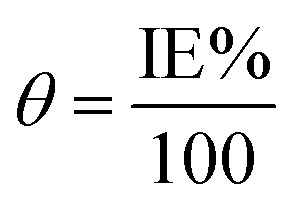
where IE%, *C*_R_ (mg cm^−2^ h^−1^), and *θ* denote the inhibition efficiency (%), rate of corrosion, and surface coverage, respectively. The rates of corrosion for the uninhibited and inhibited conditions are (*C*_R_)_a_ and (*C*_R_)_p_, respectively. *s* is the surface area in cm^2^ and *t* is the exposure duration (4 h). Δ*w* stands for the difference between the initial and final weight of the specimens at different concentrations under study.

### Electrochemical measurements

A CHI660D electrochemical workstation was employed for the electrochemical (EIS and PDP) studies. The nature of the electrodes and method used to prepare them were similar to that reported in our previous paper.^1^ After 30 min of immersion, the electrochemical experiments were performed. The establishment of an open circuit potential (OCP) necessitated almost 30 min immersion. EIS data were recorded by employing a 10 mV signal in the frequency range of 0.01 Hz to 100 kHz. By fitting the Nyquist curves of both the metallic specimens that were inhibited and uninhibited, the values for polarization resistance (*R*_p_) were obtained, and then the percentage inhibition (IE%) was calculated by using the following formula.^1,7^4
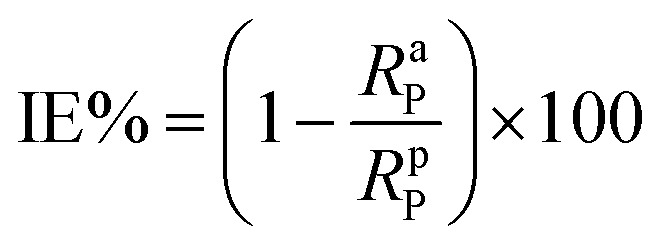
where *R*^a^_P_ and *R*^p^_P_ are the polarization resistance in the absence and presence of the synthesized inhibitor, respectively. The working electrode potentials against the corrosion potential (*E*_corr_) for the potentiodynamic polarization study were varied in the range of −250 mV to +250 mV. To determine the values of corrosion current density (*i*_corr_), the linear Tafel curve sections were extrapolated. The percentage inhibition (IE%) by using these values was calculated using eqn (5).^1,6^5
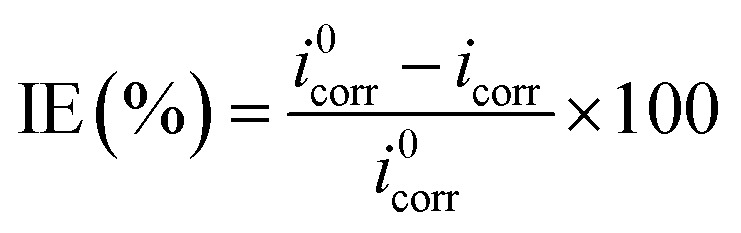
where *i*_corr_ and *i*^0^_corr_ are the corrosion current densities in the absence and presence of the inhibitor, respectively.

### Computational calculations

The density functional theory (DFT) method was employed to calculate the quantum parameters of our investigated inhibitor MISB. Optimization of the geometrical structure of the prepared inhibitor was done using Becks three-parameter exchange functional B3 with the Lee–Yang–Parr (LYP) non-local correlation functional and 6-321 + G basis set. The molecules were created using Gauss View 6.0 executed in the Gaussian 09 program package.^32,33^ Key parameters such as the energy of the highest occupied molecular orbital (*E*_HOMO_) and the energy of the lowest unoccupied molecular orbital (*E*_LUMO_), energy gap (Δ*E*) between the *E*_LUMO_ and *E*_HOMO_, electronegativity (*χ*), hardness (*η*), and softness (*σ*) fraction of transferred electrons (Δ*N*) were also calculated using the following formulas:^34,35^

6 Ionization potential (*I*) = −*E*_HOMO_

7 Electron affinity (*A*) = −*E*_LUMO_

8 Electronegativity (*χ*) = (*I* + *A*)/2

9 Electronic hardness (*η*) = (*I* − *A*)/2

10 Chemical softness (*σ*) = 1/*η*

11
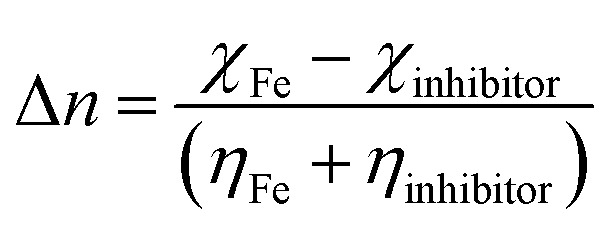


### Surface studies

To analyze the surface, cleaned and dried specimens were immersed in 100 mL of 0.5 M HCl for 4 h, both with and without the addition of MISB at the optimum concentration. Subsequently, the samples were cleaned, dried, and subjected to SEM analysis for surface morphological studies. A model X Flash Detector 5010 BRUKER Nano scanning electron microscope was used to record the SEM images of the polished, inhibited, and uninhibited specimens.

## Results and discussion

### Weight loss measurements

#### Effect of concentration

Table 1 shows the variation in IE% values with MISB molecule concentration in the acidic dissolution of mild steel. The results show that ability of MISB to protect from corrosion increased as its concentration increased, attaining the highest efficiency at 42.80 × 10^−3^ mM concentration. Careful examination of Table 1 indicated that its protective ability significantly improved as its concentration increased from 8.56 × 10^−3^ to 34.20 × 10^−3^ mM. However, there was only a slight increase in IE% when its concentration increased from 34.20 × 10^−3^ to 42.80 × 10^−3^ mM. These findings indicate that the optimum concentration of MISB is 34.20 × 10^−3^ mM. The synthesized compound could prevent corrosion because it contains enough free electrons, including a lone pair on nitrogen and electron pair on carbonyl, and these electrons are tightly bound to the positively charged metal surface.^1,12,36^

**Table 1:** Weight loss measurement parameters without and with varying concentrations of MISB inhibitor molecules

Inhibitor	*C* (mM)	*C*_R_ (mg cm^−2^ h^−1^)	IE%	*θ*
Blank		5.83		
MISB	8.56 × 10^−3^	2.41	59.08	0.58
	17.10 × 10^−3^	1.22	78.80	0.77
	25.70 × 10^−3^	0.92	84.07	0.85
	34.20 × 10^−3^	0.51	91.48	0.92
	42.80 × 10^−3^	0.07	92.07	0.91

#### Effect of temperature

**Fig. 2:**
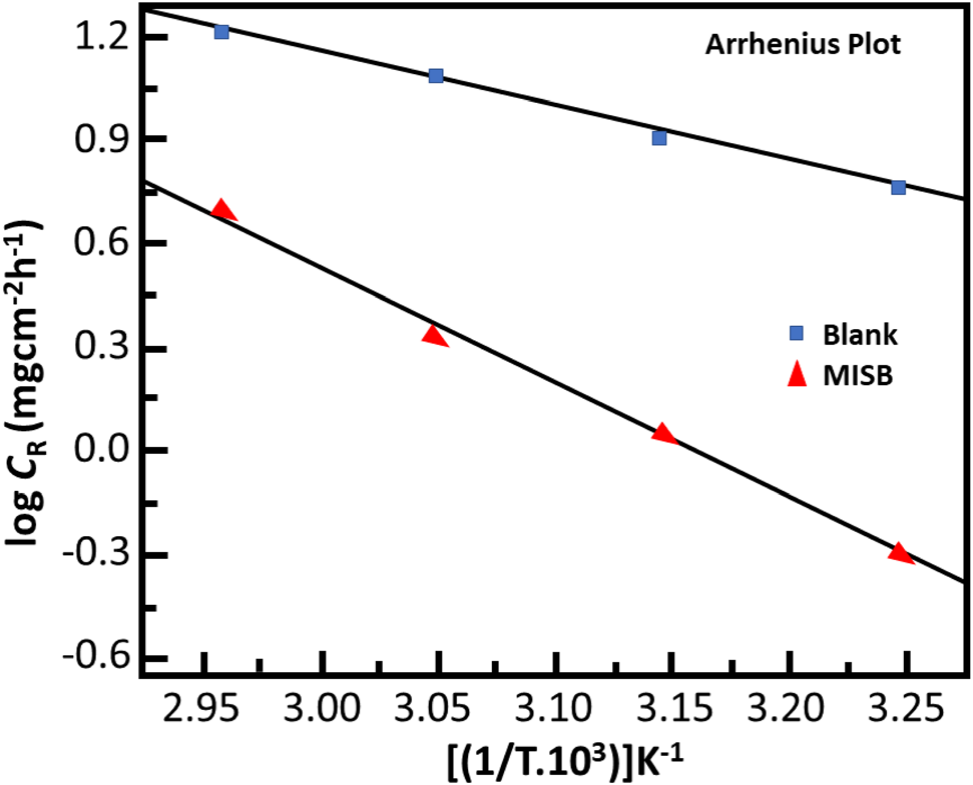
Arrhenius plots for the corrosion of mild steel in the presence and absence of MISB.

Table 2 shows the effect of temperature on the capacity of MISB to prevent the acidic dissolution of mild steel. The findings show that the inhibition efficiency of the synthetic inhibitor molecules deteriorated and a corresponding increase in the corrosion rate values with an increase in temperature. Numerous high temperature-related processes, including molecular fragmentation, acid-catalyzed molecular rearrangement, desorption and molecular etching of adsorbed MISB inhibitor molecules, are thought to be responsible for the decrease in the ability of MISB to protect against corrosion.^1,37^ The increase in kinetic energy at higher temperatures causes the desorption of the adsorbed inhibitor molecules from the surface of the metal, and consequently the force constant between the inhibitor molecules and the metal surface is reduced.^38^ The most popular method for describing the impact of temperature on the interactions of the inhibitor with metal is the Arrhenius equation, which can be written as follows:^1^12
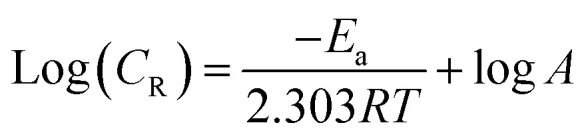
where *E*_a_, *A*, *R*, and *T* represent the activation energy, Arrhenius pre-exponential factor, universal gas constant, and absolute temperature, respectively. The slopes of the Arrhenius plots (Fig. 2) were used to calculate the *E*_a_ values. The *E*_a_ was calculated to be 65.70 kJ mol^−1^, whereas it was just 30.50 kJ mol^−1^ in the absence of MISB molecules. The adsorption and formation of a defensive barricade by the inhibitor molecules is the cause for the elevated activation energy under the inhibited conditions. By adhering to the surface, the inhibitor molecules produced a coating, which improved the energy barrier for the corrosion process.

**Table 2:** The values of rate of corrosion (*C*_R_), inhibition efficiency (IE%), adsorption constant (*K*_ads_), and Gibb's free energy (Δ*G*_ads_) for mild steel in 0.5 M HCl at different temperatures

Temperature (°C)	Blank	MISB			
	*C*_R_ (mg cm^−2^ h^−1^)	Δ*G*_ads_ (kJ mol^−1^)	*K*_ads_ × 10^4^ (L mol^−1^)	*C*_R_ (mg cm^−2^ h^−1^)	IE%
35	5.86	−34.52	1.3	0.50	91.47
45	8.20	−34.21	0.75	1.13	86.19
55	12.40	−34.53	0.56	2.17	82.52
65	16.60	−33.53	0.27	5.07	69.47

### Adsorption isotherms

**Fig. 3:**
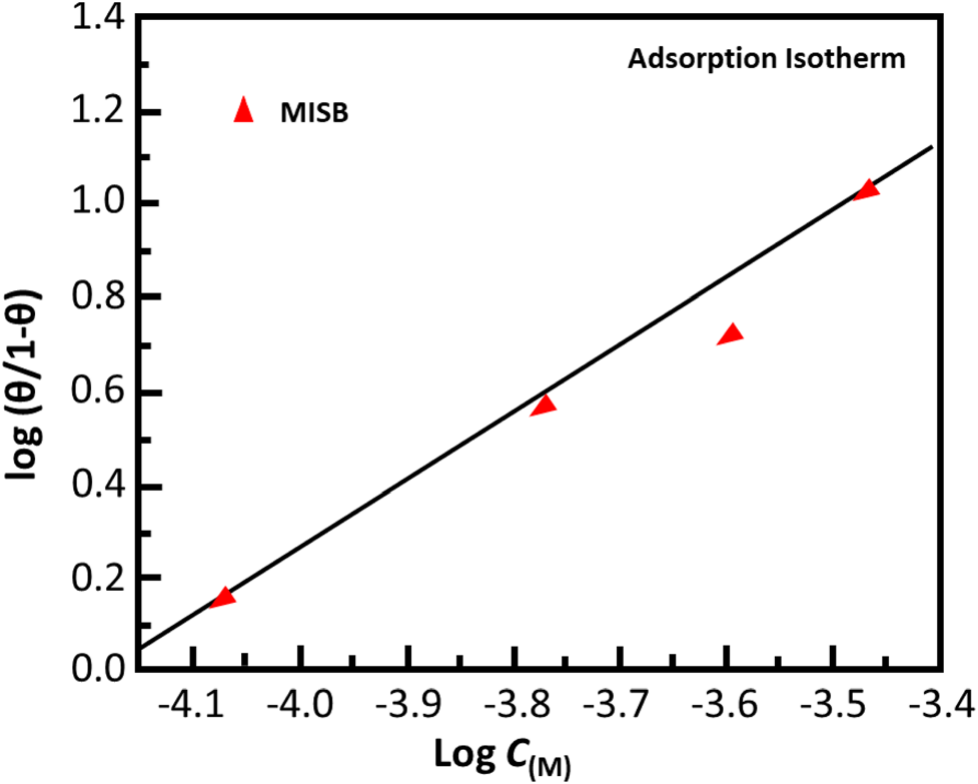
Langmuir adsorption isotherm plotted for the adsorption of MISB on mild steel surface in 0.5 M HCl.

The adsorption isotherm model serves as the most accurate representation of the MISB interactions with metallic surfaces.^1,12^ To illustrate the adsorption behavior of the synthesized MISB inhibitor on the surface, some common isotherms were tested. Fig. 3 displays the Langmuir adsorption isotherms. By using the Langmuir adsorption isotherm equation presented below, the values for the adsorption constants at the optimal concentration of MISB at various temperatures were evaluated.13
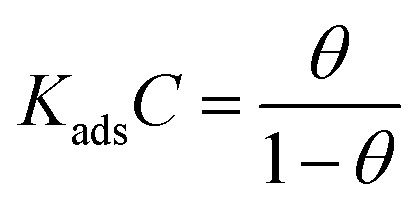
where *C* stands for the molar concentration of MISB molecules and *θ* stands for the degree of surface coverage. Typically, a high *K*_ads_ value reflects a strong absorption ability. Table 2 displays the calculated *K*_ads_ values for the various examined temperatures. The *G*_ads_ was determined for each temperature using eqn (14).14
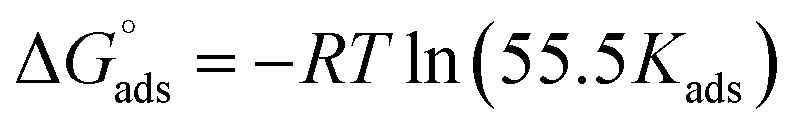
where the numeral 55.5 stands for the water concentration in acidic solution and the other symbols have their conventional meanings. Table 2 also illustrates the calculated *K*_ads_ values. As can be observed, MISB has a very high negative *G*_ads_ value, which indicates that it has a significant capacity for adsorption,^1^ and also the significant tendency of adsorption on the metallic surface was indicated by the high values of *K*_ads_ for MISB.

### Open circuit potential test

**Fig. 4:**
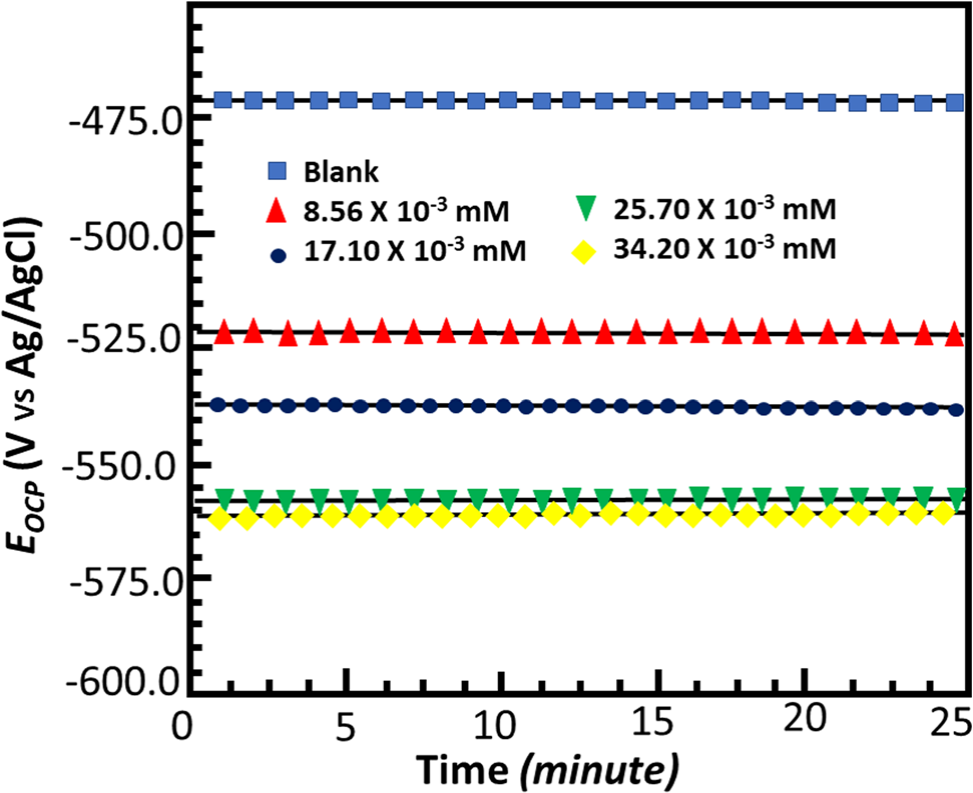
Open circuit potential *vs.* time curve for corrosion of mild steel in 0.5 M HCl in the absence and presence of various concentrations of MISB molecules.

To support the weight loss experiment, electrochemical measurements were conducted. The open circuit potential is the difference between the potentials of the working electrode and the standard or reference electrode when no external current is applied. Fig. 4 displays the open circuit potential *versus* time curves for 25 minute curves following 30 min immersion. As can be observed, the OCP *vs.* time curves for the inhibited and uninhibited conditions depict straight lines, indicating that a steady-state potential developed in both cases. The straight lines also show that the Fe_2_O_3_ and Fe_3_O_4_ oxide layers were entirely eliminated, and that a protective or inhibitive film by the MISB inhibitor was formed on the metal surface. Additionally, it can be observed that the open circuit potential *vs.* time curves shifted with MISB in the cathodic or negative direction. This result indicates that although the presence of MISB affects both the cathodic and anodic processes, its effectiveness towards cathodic reactions is comparatively higher due to the precipitation of the inhibitors molecule on the cathodic sites of the metal surface.

### Potentiodynamic polarization studies

**Fig. 5:**
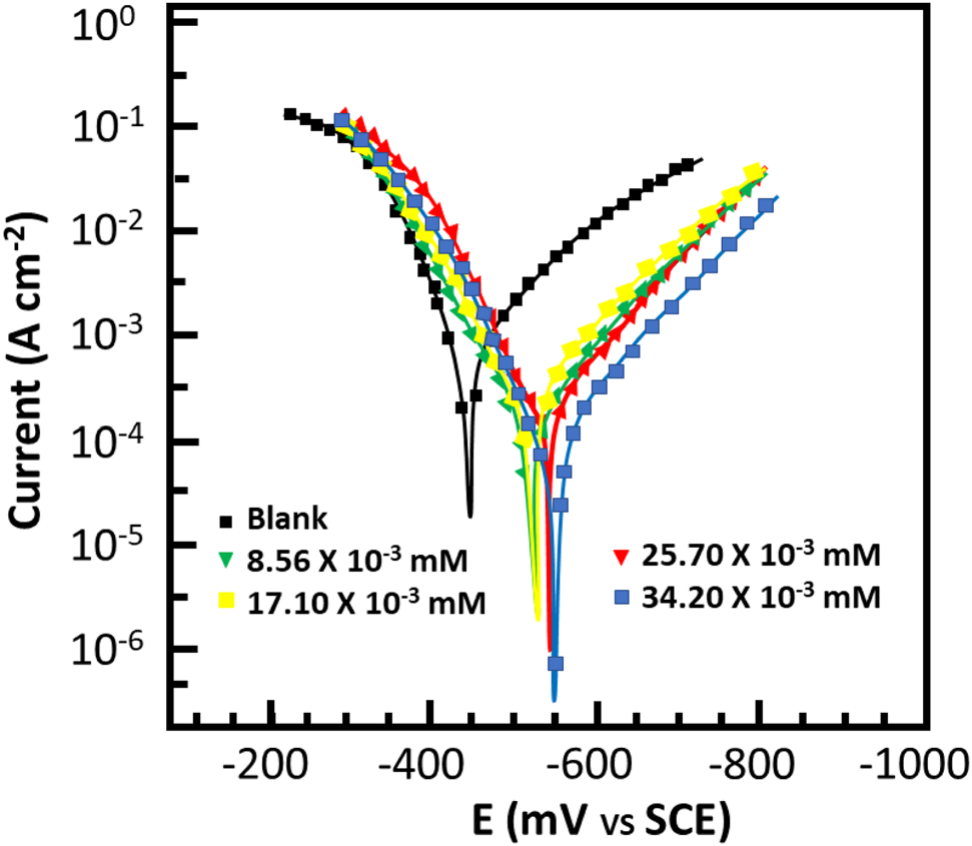
Polarization curve of mild steel in 0.5 M HCl with and without MISB molecules.

For thorough insight into the inhibitor behavior toward the anodic and cathodic processes, potentiodynamic polarization studies were conducted. Fig. 5 depicts the cathodic and anodic polarization curves for metallic dissolution in 0.5 M HCl with and without MISB, and Table 3 lists the polarization indices of the inhibitor. Obviously, at the various concentrations tested, MISB had an impact on both the cathodic and anodic reactions and processes, causing a significant drop in the corrosion current density (*i*_corr_) without altering the typical Tafel curve appearance. This observation reveals that the MISB molecules hinder the process of corrosion by blocking the surface-active sites through adsorption.^39^ This result implies that the examined inhibitor precipitated over the cathodic region, and subsequently behaved as mostly a cathodic-type inhibitor given that the corrosion potential (*E*_corr_) in the Tafel curve inhibited by MISB shifted towards the negative site.^40^ The shift in the value of the corrosion potential of the inhibited Tafel curve relative to the uninhibited Tafel curve can be used to characterize the cathodic and/or anodic nature of the synthesized inhibitor.

**Table 3:** Polarization parameters for the corrosion of mild steel in 0.5 M HCl without and with various concentrations of MISB molecules

Inhibitor	*C* (mM)	*E*_corr_ (mV per SCE)	*β*_a_ (μA cm^−2^)	*β*_c_ (mV dec^−1^)	*i*_corr_ (μA cm^−2^)	IE%	*θ*
Blank		−445	70.6	114.8	1130		
MISB	8.56 × 10^−3^	−522	69.2	107.8	462.0	59.21	0.60
	17.10 × 10^−3^	−545	164.7	146.3	229.0	79.83	0.80
	25.70 × 10^−3^	−525	72.3	76.4	173.0	84.78	0.83
	34.20 × 10^−3^	−541	132.6	152.1	97.0	91.51	0.92

### Electrochemical impedance spectroscopy

**Fig. 6:**
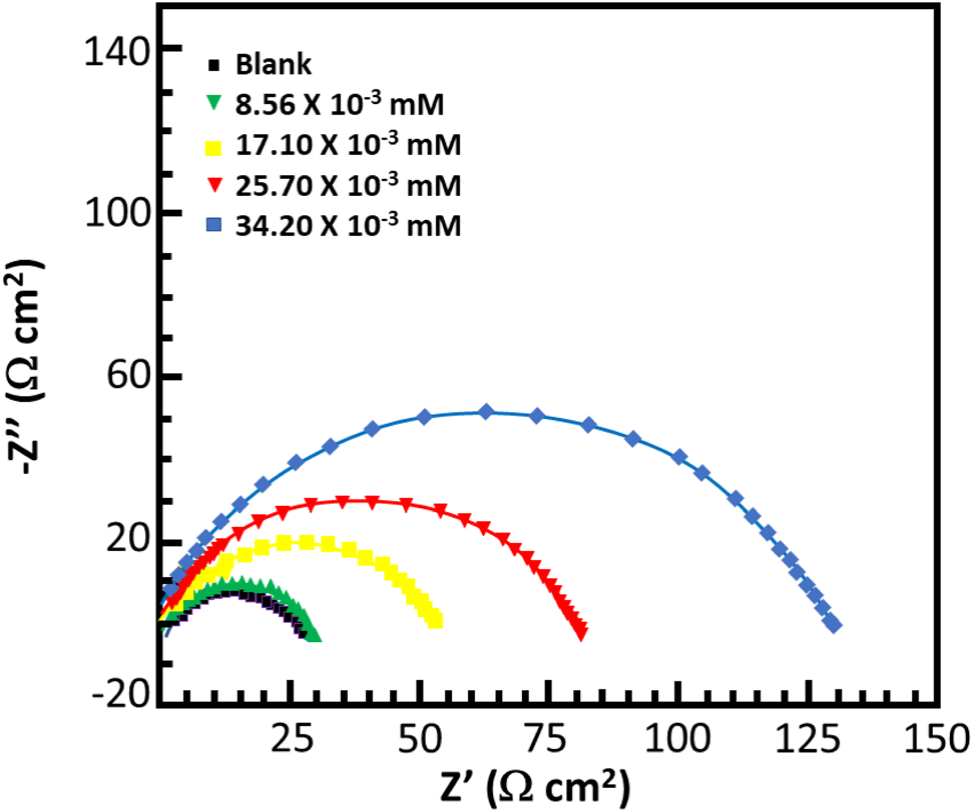
Nyquist plots for corrosion of mild steel in 0.5 M HCl without and with various concentrations of MISB molecules.

**Fig. 7:**
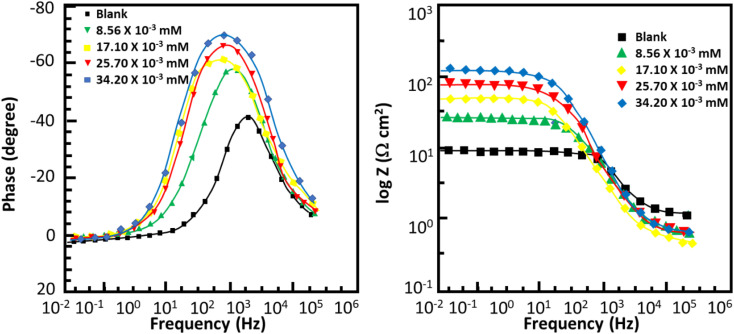
Bode plots for corrosion of mild steel in 0.5 M HCl without and with various concentrations of MISB molecules.

Fig. 6 and 7 show the Nyquist and Bode plots for the corrosion of mild steel in acidic conditions with and without MISB molecules, respectively. At all concentrations under investigation, the Nyquist plots appear as a single semicircle, which suggests a single charge transfer reaction. It is evident that the diameters of the semicircles in the Nyquist plots are larger for the inhibited metallic specimens than for the uninhibited metallic specimens (blank). Additionally, at higher concentrations of MISB, the diameter of the semicircle increased more noticeably. The Nyquist plots of the metallic samples under the inhibited and uninhibited conditions were fitted in the suitable equivalent circuit to derive various impedance characteristics. In the described circuit, a constant phase element (CPE) was used in place of a pure capacitor given that it gives a greater understanding of the interactions between the metal and electrolyte at interfaces. Subsequently, we present the impedance of the constant phase element, which is typically indicated by *Z*_CPE_.

15 *Z*_CPE_ = *Y*_0_^−1^((i*ω*)^*n*^)^−1^

where *ω*, *Y*_0_, *n*, and i represent the angular frequency, CPE constant, phase shift, and an imaginary number, respectively. Given that a high value of *n* correlates with a high level of surface smoothness and *vice versa*, it can also be used as a measure of surface roughness or smoothness.^41^Table 4 lists the calculated impedance parameters, % inhibition efficiencies, and surface coverage. The results show that, with a few exceptions, the *n* values are higher in the inhibited solution than in the uninhibited condition. This discovery shows that the metallic surface was substantially smoother in the presence of MISB, especially at a greater concentration, than in its absence. Additionally, the *n* values for both the inhibited and uninhibited conditions are close to unity, showing that the CPE functioned as a pseudo-capacitor during the investigation. Organic inhibitors are thought to be able to suppress corrosion in aggressive acidic media by adsorption on metal–electrolyte interfaces, which leads to the formation of an electric double layer.^1,42^ The findings in Table 4 demonstrate that the *R*_ct_ values for the inhibited case are much greater than that for the non-inhibited condition. This result suggests that the difficulty of charge transfer in the presence of MISB is due to its adhesion to the metal–electrolyte interfaces. According to additional findings, the increase in *R*_ct_ value is much more noticeable at higher inhibitor concentrations.

**Table 4:** EIS parameters for the corrosion of mild steel in 0.5 M HCl without and with various concentrations MISB inhibitor molecules

Inhibitor	*C* (mM)	*R*_s_ (Ω cm^2^)	*R*_p_ (Ω cm^2^)	*n*	IE%	*θ*
Blank		1.13	10.71	0.828		
MISB	8.56 × 10^−3^	0.727	26.80	0.812	60.06	0.61
	17.10 × 10^−3^	0.520	51.19	0.816	79.10	0.80
	25.70 × 10^−3^	0.693	78.01	0.843	82.29	0.83
	34.20 × 10^−3^	0.569	127.21	0.856	91.60	0.93

### Computational studies

**Fig. 8:**
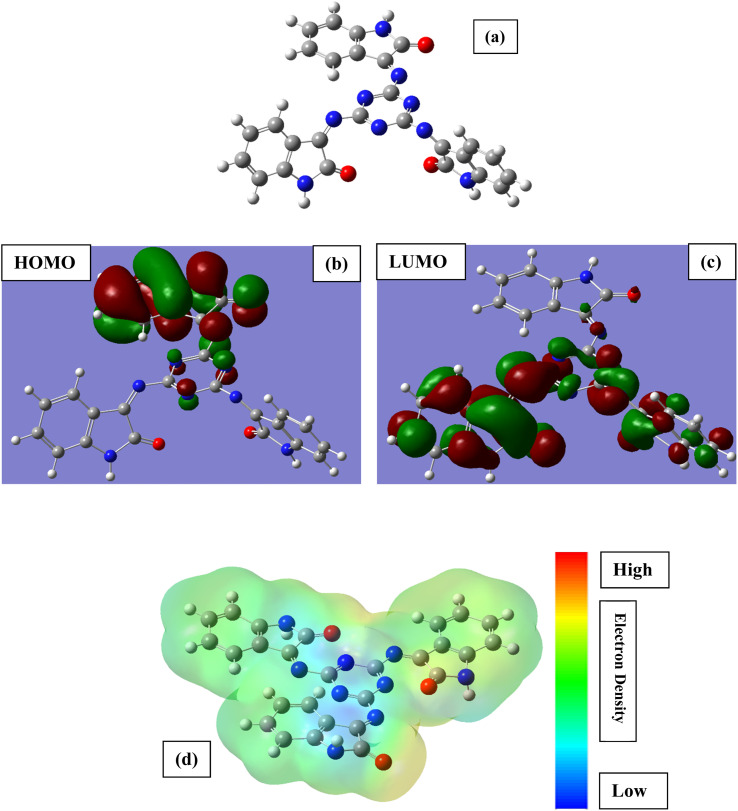
Optimized chemical structure (a), highest occupied molecular orbital (b), lowest unoccupied molecular orbital (c) and molecular electrostatic potential (d) of the tested inhibitor MISB.

Fig. 8 depicts the frontier molecular orbitals (optimized, HOMO, LUMO and MEP) of MISB, while Table 5 lists the calculated DFT indices. The DFT parameters in the table are in good agreement with the experimental data. Generally, the interactions of corrosion inhibitors on a metallic surface are comprised of donor–acceptor bonding, where in these interactions, *E*_HOMO_ is associated with the capacity of the inhibitor to transmit electrons (charge), where *E*_LUMO_ is associated with its ability to accept electrons. Hence, strong metal–inhibitor binding and good protective ability are related with high values of *E*_HOMO_ and low values of *E*_LUMO_, respectively.^43–47^ The highest occupied-molecular-orbital (*E*_HOMO_) energy shows the capability of the tested inhibitor to donate electrons. The capability of the molecules to accept electrons from the back donation of iron, and to thus enhance the binding energy between the metal and the inhibitor is shown by a lower *E*_LUMO_ value. The greater the *E*_HOMO_ and the lower the *E*_LUMO_, the better the capacity of the tested inhibitor to attach to the metal surface.^3–5,30^ This suggests that MISB is an efficient corrosion inhibitor, which is in good agreement with the experimental findings.^48^ The chemical response was determined by the energy gap (*E*_gap_) values. In terms of reactivity, the more reactive the molecule towards the substrate surface, the higher its inhibitory efficiency, and the smaller the Δ*E* gap, the more stable it is. Consequently, the combination of MISB with the Fe substrate was stable. The dipole moment (*μ*) is caused by the nonuniform surface charge distribution of the atoms in the molecules. A low dipole moment value supports the capacity of a molecule as an inhibitor. According to the hard–soft acid–base concept, a soft molecule in has a lower *E*_gap_ value and greater basicity, whereas the opposite is observed for hard molecules.^49^ Consequently, a soft molecule has greater adsorption ability due to its easier electron transfer and it is a stronger corrosion inhibitor than a hard molecule.

**Table 5:** DFT parameters for neutral and protonated form of MISB inhibitor molecule

MISB	*E*_HOMO_ (eV)	*E*_LUMO_ (eV)	Δ*E* (eV)	*η* (eV)	*σ* (eV)	*χ* (eV)	Δ*N* (eV)	*μ* (debye)
Neutral	−0.21	−0.09	0.15	0.06	16.66	0.12	0.207	6.32

According to Lukovit's research, when the number of electrons transmitted (Δ*N*) is less than 3.6, the inhibition performance improves as a function of the electron transfer.^49^ The larger the fraction of electron transport (Δ*N*), the better the corrosion inhibitor. Thus, the high value of Δ*N* for MISB suggests that it is a good corrosion inhibitor and electron donor.

The global electronegativity value for MISB was also computed. A high global electronegativity (*χ*) value suggests that the investigated compound is less potent to donate/transfer its electron to the appropriate acceptor molecule, *e.g.*, d-orbital of the surface Fe atoms in the present case.

MISB shows an electronegativity value of 0.12. The low value of *χ* for MISB indicates that it has high electron transfer ability, and thereby acts as good corrosion inhibitor. Based on the values of *E*_LUMO_ and *E*_HOMO_, the global hardness (*η*) and softness (*σ*) values were also derived for the MISB molecule. A high value of *σ* is related with high reactivity; electron donating ability, adsorption tendency and inhibition efficiency, while the opposite is observed for *η*.^49,50^ The results show that MISB has high *σ* value (16.66 eV) and low *η* value (0.66 eV), which suggest that MISB is a highly reactive and potent corrosion inhibitor. Based on the above discussion, it can be concluded that the DFT study provided good support for the experimental results and findings.

### Suggested corrosion-inhibition mechanism

The inhibitory inertia of organic molecules is caused by the formation of a protective layer that is adsorbed onto the iron surface. Gravimetric measurements revealed that the tested inhibitor greatly reduced the corrosion of mild-steel. Moreover, the adsorption isotherm studies revealed that the investigated inhibitor molecules adhered to the mild-steel surface and follow the Langmuir adsorption model. Furthermore, the adsorption behavior of the generated protective film is principally determined by the following: (1) electrostatic interactions through protonated heteroatoms and (2) different linkages between inhibitor molecules and the mild-steel surface.^51^

**Fig. 9:**
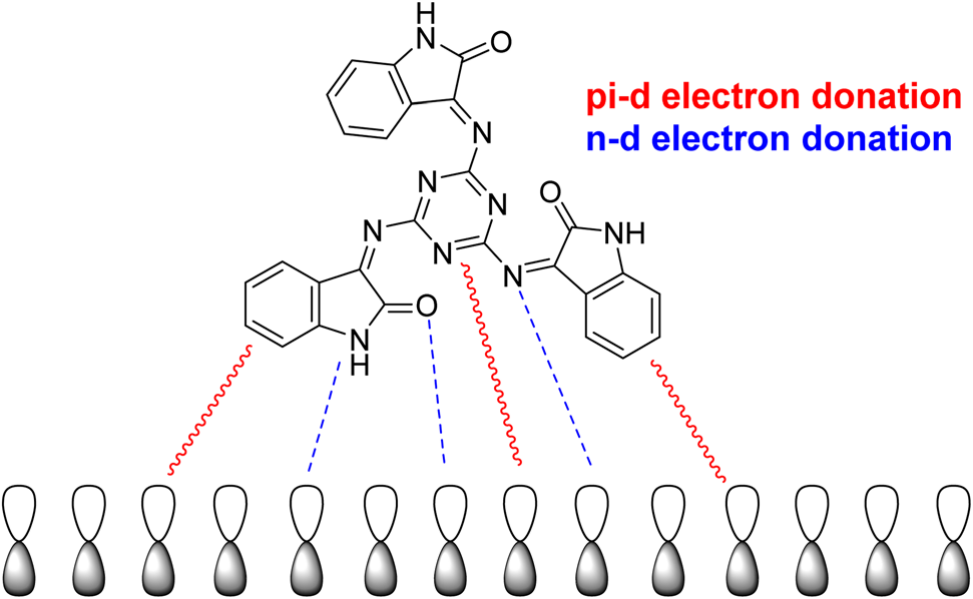
Suggested corrosion-inhibition mechanism of mild steel in 0.5 M HCl with the addition of the examined inhibitor (MISB).

Fig. 9 illustrates in further detail how the tested inhibitor compounds interact with the mild-steel surface. The most common route of adsorption between the inhibitor molecule and the mild-steel surface involves the interaction of both the pi-electrons of the aromatic rings and the vacant d-orbital of the metal atoms. The second method involves interactions between the lone electron pairs in the heteroatoms and the unoccupied d-orbitals on the surface of iron (mild steel) atoms. The d-orbitals of the Fe atom will share these active electrons.

### Morphological investigation

**Fig. 10:**
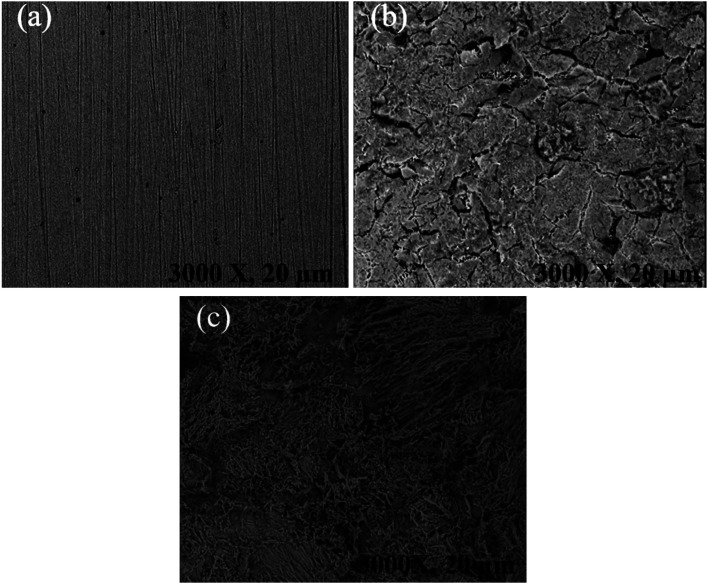
SEM image of mild steel specimen for 4 h immersion time in 0.5 M HCl (a) before immersion, (b) immersed in 0.5 M HCl without inhibitor and (c) immersed in 0.5 M HCl with MISB inhibitor molecules.

Fig. 10 displays the SEM images of the mild steel specimen that was corroded for 3 h. As can be seen from the extremely rough surface and visible pits and fissures, the unprotected metallic specimens in the SEM photos are severely corroded and damaged. However, as can be seen from the SEM image, the metallic surface greatly improved in the presence of the synthesized MISB inhibitor. It can be postulated that MISB generated surface films, which shield the metallic surfaces from corrosion, based on the better surface morphologies of the shielded metallic specimens. This result also confirmed the pattern of inhibitory effectiveness attained through the weight loss and electrochemical techniques.

## Conclusion

In conclusion, we showed the synthesis of a melamine–isatin hybrid tris-Schiff base (MISB) and investigated its potential to function as a mixed-type inhibitor. The synthetic inhibitor demonstrated a high mild steel inhibition performance in 0.5 M HCl. According to the evaluation of weight loss, the efficacy of inhibition increased as the inhibitor concentration increased and it was reduced as the temperature increased, and the maximum efficiency of 92.06% was derived for MISB. According to the electrochemical impedance measurements, the increased inhibition efficacy of the inhibitor solution was caused by its high charge transfer resistance. The Langmuir-type isotherm was identified through the analysis of the adsorption isotherms and thermodynamic parameters, indicating physisorption. The polarization analysis revealed that MISB functions primarily as a cathodic-type inhibitor. Further evidence for the ability of the synthetic tris-Schiff base to suppress corrosion was provided by SEM images, which showed the development of a protective layer on the surface of mild steel.

## Conflicts of interest

There are no conflicts to declare.

## Supplementary Material

RA-013-D3RA00357D-s001
