# Supplementary material for: Melamine–isatin tris Schiff base as an efficient corrosion inhibitor for mild steel in 0.5 molar hydrochloric acid solution: weight loss, electrochemical and surface studies
Source: RSC Adv. 2023 Jun 26;13(28):19301–11. doi: 10.1039/d3ra00357d (PMC10291281; doi:10.1039/d3ra00357d)
Supplement: RA-013-D3RA00357D-s001 [file RA-013-D3RA00357D-s001.pdf]

Electronic Supplementary Material (ESI) for RSC Advances.
This journal is © The Royal Society of Chemistry 2023

Melamine-Isatin Tris Schiff Base as An Efficient Corrosion Inhibitor for Mild Steel in 0.5 Molar Hydrochloric Acid Solution: Weight loss, Electrochemical and Surface Studies

Ifzan Arshad[a]*, Khizer Qureshi[a], Awais Siddique Saleemi[b], Ali Abdullah[b], Aboud Ahmed awadh Bahajjaj[c], Shafaqat Ali[d,e], Awais Ahmed[f]

[a] Department of Chemistry, University of Management and Technology, Sialkot, Pakistan

[b] Nano & Micro FT Laboratory, Program of Physics, University of Management and Technology, Sialkot Campus, Sialkot, 51310, Pakistan

[c] Department of Chemistry, College of Science, King Saud University, Riyadh 11451, Saudi Arabia

[d] Department of Environmental Science, Government College University Faisalabad, Faisalabad 38000, Punjab, Pakistan

[e] Department of Biological Science and Technology, China Medical University, Taichung, 40402, Taiwan

Departamento de Química Orgánica, Universidad de Córdoba, Campus de Rabanales, Edificio Marie Curie (C-3) Ctra Nnal IV, Km 396, Córdoba (Spain) E-14014

Corresponding author. Ifzan Arshad, *E-mail address*: arshadmi@mail.ustc.edu.cn

Abstract

In the current study, the 3,3',3"-((1,3,5-triazine-2,4,6-triyl)tris(azaneylylidene))tris(indolin-2-one) (MISB) condensation product of melamine (triazine) and isatin is investigated as a mild steel corrosion inhibitor in a 0.5 M HCl. The ability of the synthesized tris-Schiff base to suppress corrosion was evaluated utilizing weight loss measurements and electrochemical techniques. The maximum inhibition efficiency of 92.07 %, 91.51 % and 91.60 % were achieved using 34.20 x 10^{-8} mM of MISB in weight loss measurements, polarization, and EIS tests, respectively. It was revealed that temperature rise decreased inhibition performance while increasing inhibitor concentration increased it. The analyses demonstrated that the synthesized tris-Schiff base inhibitor followed the Langmuir adsorption isotherm and was an effective mixed-type inhibitor having a low cathodic

predominance. According to the electrochemical impedance measurements, the R_{ct} values increased with the increase of inhibitor concentration. The weight loss and electrochemical assessments was also supported by surface characterization analysis and SEM images shows a substantial smoothness in the surface morphology.

Keywords Corrosion, inhibition, Mild Steel, Electrochemical, Synthesis

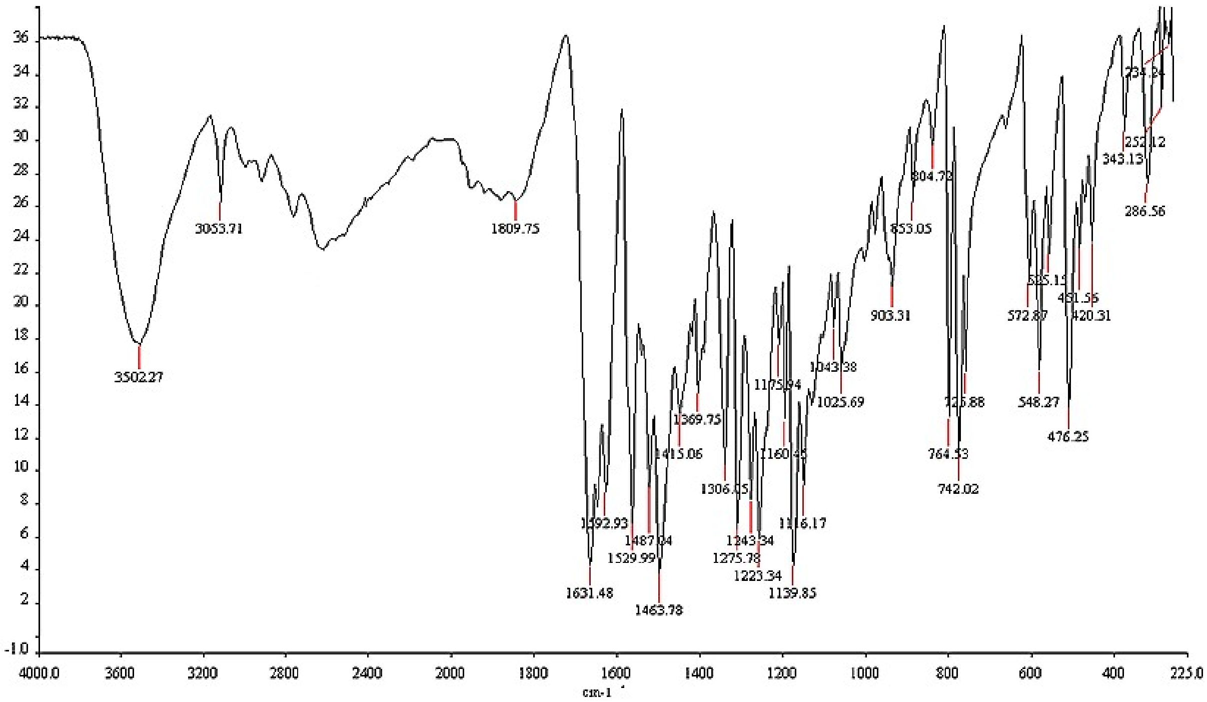

Fig.1 FTIR Spectra of **MISB**

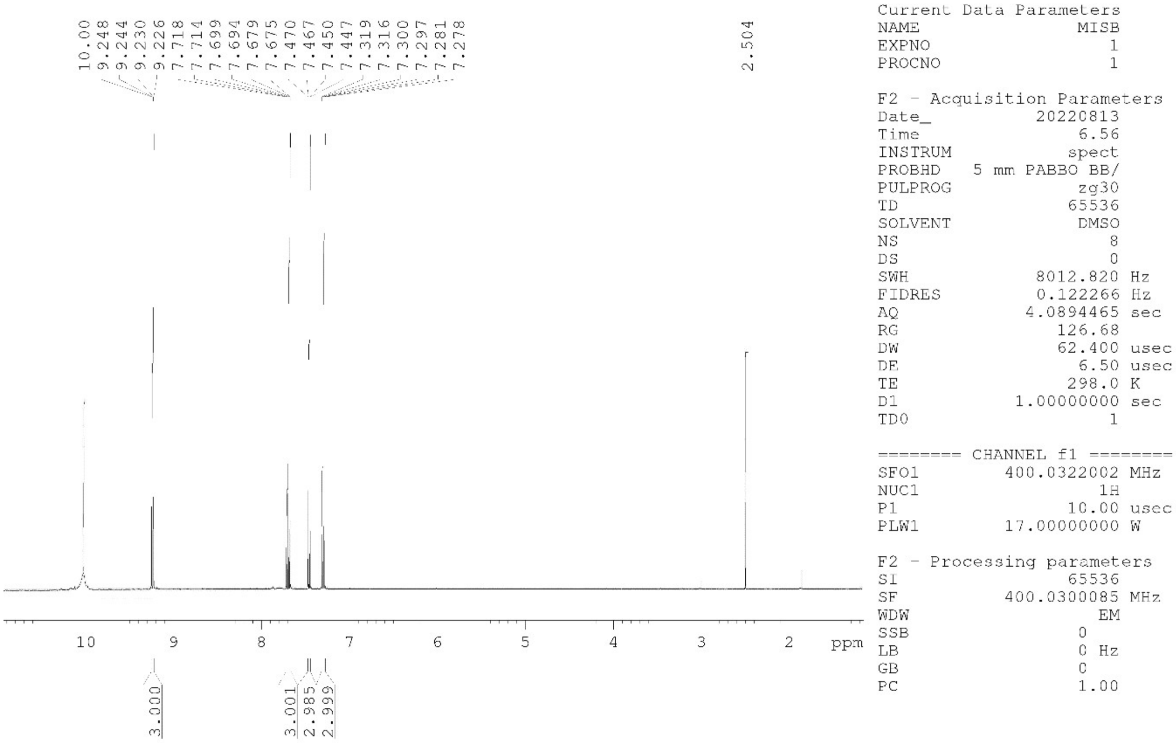

Fig.1 ^{1}H NMR of **BNSB01**

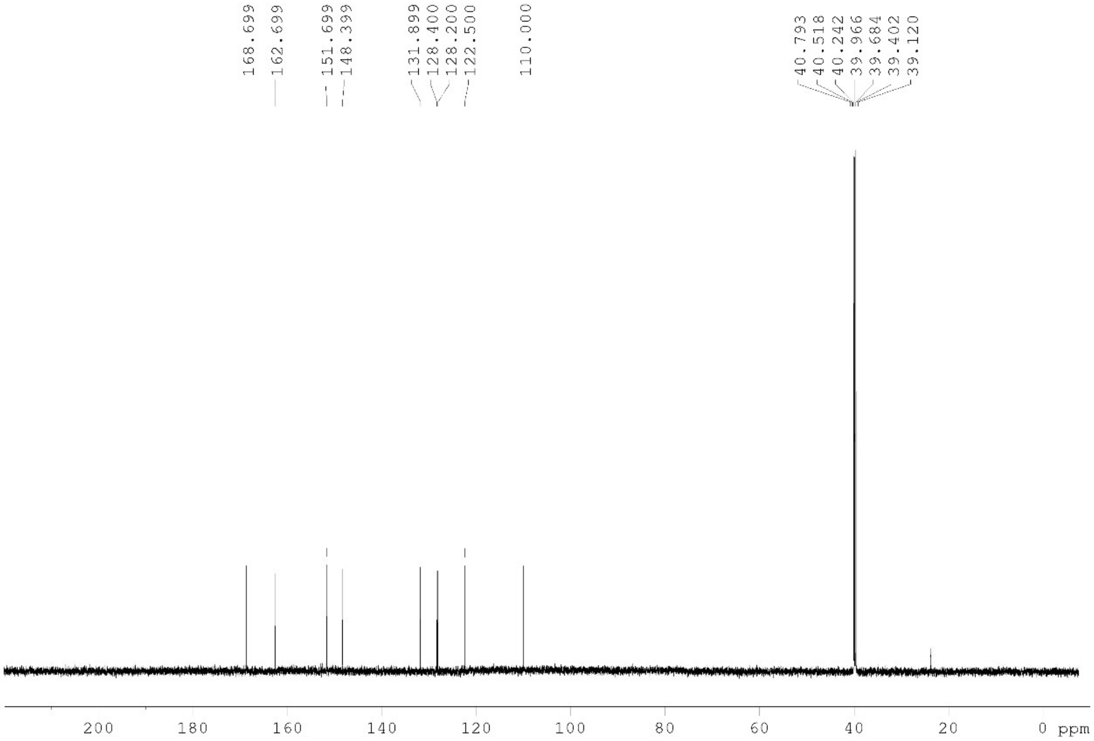

Fig.2 ^{13}C NMR of **BNSB01**
